# Evolution of Pediatric Home Mechanical Ventilation Program in Serbia—What Has Changed in the Last Decade

**DOI:** 10.3389/fped.2020.00261

**Published:** 2020-06-10

**Authors:** Mihail Basa, Predrag Minic, Milan Rodic, Aleksandar Sovtic

**Affiliations:** ^1^Department of Pulmonology, Mother and Child Health Care Institute, Belgrade, Serbia; ^2^School of Medicine, University of Belgrade, Belgrade, Serbia

**Keywords:** home mechanical ventilation, non-invasive ventilation, tracheostomy, chronic respiratory failure, alveolar hypoventilation, obstructive sleep apnea

## Abstract

Home mechanical ventilation (HMV) is a method of treatment in children with sleep-disordered breathing (SDB) and alveolar hypoventilation regardless of primary disease. The goal of the study was to describe the changes in the HMV program in Serbia during the last two decades. Cross-sectional retrospective study included data from the national HMV database from 2001 until 2019. HMV was initiated in clinically stable patients after the failure to wean from mechanical ventilation succeeded acute respiratory deterioration or electively after the confirmation of SDB and alveolar hypoventilation by sleep study or continuous transcutaneous capnometry and oximetry. The study included 105 patients (50 ventilated noninvasively and 55 ventilated invasively via tracheostomy). The median age at the time of HMV initiation was 6.2 years (range: 0.3–18 years). Invasive ventilation had been initiated significantly earlier than noninvasive ventilation (NIV) (*p* < 0.01), without difference in duration of ventilatory support (*p* = 0.95). Patients on NIV were significantly older (*p* < 0.01) than those ventilated invasively (13 and 1.5 years, respectively). Average waiting time on equipment had been shortened significantly—from 6.3 months until 2010 to 1 month at the end of the study (*p* < 0.01). Only 6.6% of patients had obstructive sleep apnea syndrome (OSAS) requiring HMV. During the study period, 24% patients died, mostly due to uncontrolled infection or progression of underlying disease. Availability and shortened waiting time for the equipment accompanied by advanced overall health care led to substantial improvements in the national HMV program. However, future improvements should be directed to systematic evaluation of SDB in patients with OSAS, early diagnosis of nocturnal hypoventilation, and subsequent timely initiation of chronic ventilation.

## Introduction

Mechanical ventilation at home (HMV) is a recognized method for the treatment of the sleep-disordered breathing (SDB) and alveolar hypoventilation in childhood. The population of children requiring HMV is growing rapidly worldwide, mostly as a consequence of advanced life support for technology-dependent patients and early recognition of alveolar hypoventilation (1, 2). Despite the significant financial burden on the health care system, initiation of HMV has hugely facilitated health care of these patients. Besides the obvious benefits such as life expectancy, many studies confirmed that HMV enhanced quality of life, and social interactions, along with decelerating lung function decline and improving nutritional status (3, 4). In some patients, however, HMV caused procedure-related complications (e.g., midfacial hypoplasia and malocclusion) (1, 2).

Even though the HMV trend began in high-income countries, the last decade showed that well-organized respiratory units in referral hospitals from low- and middle-income countries were capable of implementing adequate national HMV programs (5–10). The first national survey on HMV in developing countries, particularly in Serbia, was published almost a decade ago (11), following which the number of patients has increased remarkably.

The primary goal of this study was to outline the changes in the last decade regarding clinical practice and the obstacles for adoption and implementation of the HMV program in our system with limited resources. In addition, we speculated that delayed initiation of HMV and non-existence of home-care providers resulted in higher prevalence of invasively ventilated children.

## Materials and Methods

In a relatively small country such as Serbia, with seven million inhabitants (2.8 million being younger than 18 years of age), there are five university children's hospitals. The Mother and Child Health Care Institute is a multidisciplinary pediatric center and the only national HMV center. According to the national health care policy, the decision for initiation of HMV should be made in the Institute, based on careful evaluation of the medical records, patient's clinical status, and the results of diagnostic procedures.

This cross-sectional study included retrospectively evaluated medical records extracted from the Serbian national HMV database, collected from 2001 until September 2019. Data included patient demographics, underlying disease, and criteria for HMV initiation in patients who had been ventilated at home for a period of at least 3 months. Finally, the outcome was recorded—duration of HMV and reason for its termination. The study protocol was approved by the local ethics committee (decision number 8/3).

### Criteria for Initiation of HMV

The primary indication for initiation of HMV was hypercapnic respiratory failure, documented by increased PaCO_2_. Many patients had HMV started after an acute respiratory event and subsequent weaning failure, while in other cases, a sleep study was performed to document SDB. During the last 5 years, HMV was started electively after confirmation of nocturnal hypoventilation that precedes chronic respiratory failure (CRF).

Clinical tests of respiratory function with the comparison of the obtained results and monitoring of its values in order to detect potential declines were performed in patients with neuromuscular disorders (NMDs) in whom the test was possible. Peak cough flow, spirometry, and respiratory muscle strength measurements were obtained in patients who were cooperative during the procedures. Although these tests are not precisely validated, significant negative trends or low absolute values (peak cough flow < 160 L/min, forced vital capacity < 70% predicted, maximal inspiratory pressure < −60 cmH_2_O, or sniff nasal inspiratory pressure < 40 cmH_2_O) led to further evaluation of nocturnal hypoventilation. Right from the national HMV program initiation, diagnostic polygraphy was used as a screening test in patients with NMD, and all patients with a suspected OSAS apnea–hypopnea index (AHI) of >5 episodes h^−1^ were confirmed to have SDB. In addition, until 2015, continuous pulse oximetry followed by arterial blood gases analysis (ABG) after awakening was performed. From 2015 onward, diagnostic evaluation was upgraded by overnight gas exchange estimation when the equipment for continuous transcutaneous capnometry (PtcCO_2_) and oximetry became available (12). More than 2% of total sleep time (TST) with SpO_2_ < 90% or >2% of TST with PtcCO_2_ > 50 mmHg was consistent with findings of nocturnal hypoventilation (13).

Further treatment depended on primary etiology or diagnostic group. Patients with SDB and OSAS, due to upper airway obstruction, were started on continuous positive airway pressure (CPAP) or bilevel positive airway pressure (BiPAP). Patients with CRF were started on either chronic invasive ventilation or non-invasive ventilation (NIV). Our multidisciplinary team discussed every case and prescribed further treatment. Taking into consideration the objective criteria and psychosocial circumstances, each patient and his/her family was evaluated very carefully, and patients motivated and capable of starting with HMV gave their written informed consent.

For NMD patients who needed ventilation 24 h a day, either Vivo 50® (Breas) or Trilogy 100® (Respironics) ventilators were recommended. In most of these patients, tracheostomy was performed due to bulbar symptoms or weaning failure in intensive care unit (ICU). For those patients who did not require continuous ventilation, Vivo 40® (Breas) or A 40® (Respironics) ventilators had been used mostly. CPAP devices were recommended in all patients with OSAS and documented SDB. Eligible patients and their families were advised to start HMV regardless of the recommended list of indications covered by the health care system reimbursement policy. Costs of equipment were covered by parents, charities, or the national health insurance fund. When necessary, cough-assist machines were also bought by patients on their own. In majority of cases, caregivers had been the patient's parents or relatives.

### Statistical Analyses

Statistical data were analyzed by IBM SPSS Statistics 25 for Windows and are expressed as median and range. Differences in medians between non-normally distributed variables were analyzed by Kruskal–Wallis and Mann–Whitney *U*-test. Differences between categorical variables were tested using chi-square and Fisher's exact test. Correlations were evaluated by Spearman's rank correlation test. Statistically significant differences were documented by *p* < 0.05.

## Results

### Patient's Characteristics

**Figure 1:**
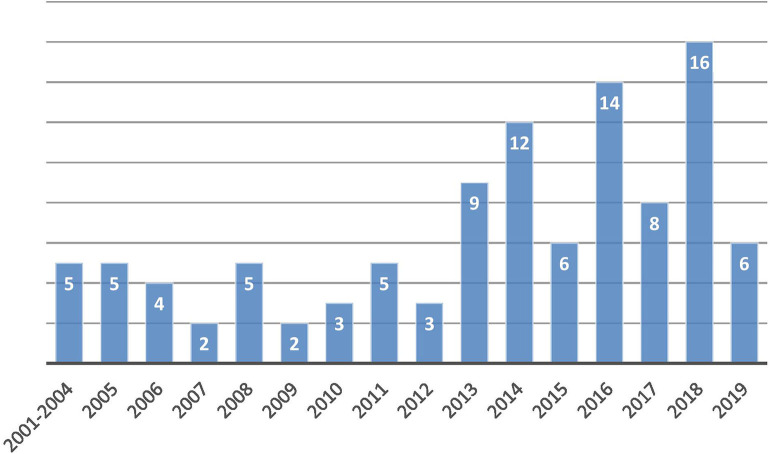
Number of new patients per year.

**Figure 2:**
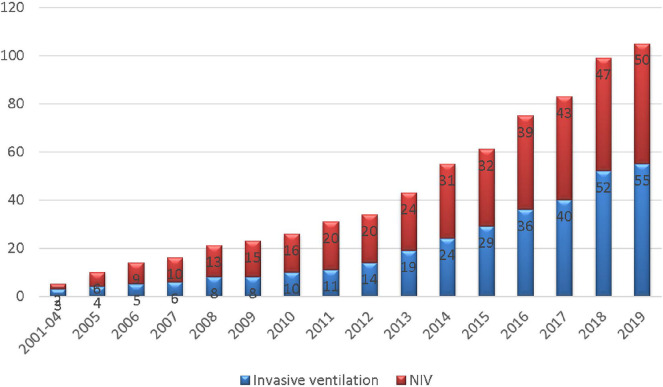
Total number of patients.

Since 2001, the number of patients has increased steeply, particularly during the last 5 years (Figure 1). The study recruited 114 patients, but 105 patients were included in the analysis (50 ventilated noninvasively and 55 ventilated invasively via tracheostomy) with equal gender distribution (Table 1, Figure 2). The study excluded nine patients for whom indication for the NIV initiation was established, but the ventilation had not been initiated due to lack of cooperation. The median age at the time of HMV initiation was 6.2 years (range: 0.3–18 years) with median age of 13 years in the NIV group and 18 months in the group ventilated invasively (*p* < 0.01). Although this difference is highly statistically significant, severe initial clinical presentation and rapid deterioration were met at an early age in most of the tracheotomized patients.

**Table 1:** Demographic data.

	**Non-invasive ventilation**	**Invasive ventilation**	**Total**
Number of subjects	50	55	105
Sex (M/F)	28/22	27/28	55/50
**Underlying disease**			
NMD	28	51	79
Primary respiratory diseases	12	2	14
CCHS	3	2	5
OSAS	7	–	7
Age at initiation of HMV (median in years with range)	13 (0.75–18)	1.5 (0.3–17.9)	6.2 (0.3–18)
Duration of HMV (median in months)	42 (3–180)	36 (3–216)	38 (3–180)
Overnight ventilation	49	12	61
Ventilation 24 h a day	1	43	44
Died	10	15	25

*NMD, neuromuscular disease; CF, cystic fibrosis; CCHS, congenital central hypoventilation syndrome; OSAS, obstructive sleep apnea syndrome; HMV, mechanical ventilation at home*.

**Figure 3:**
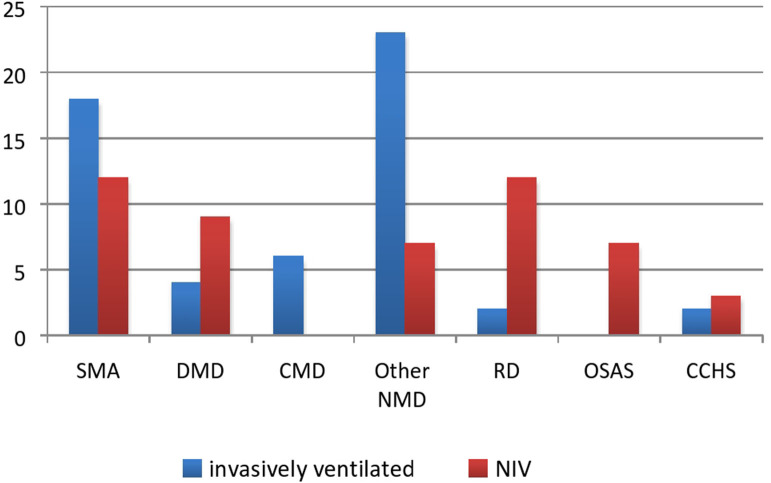
HMV strategy in each group. SMA, spinal muscular dystrophy, DMD, Duchenne muscular dystrophy, CMD, congenital myotonic dystrophy; other NMD, other neuromuscular disease; PRD, primary respiratory disease; OSAS, obstructive sleep apnea syndrome; CCHS, congenital central hypoventilation syndrome.

Patients were classified into four categories: NMDs, OSAS, syndrome of congenital central hypoventilation (CCHS), and primary respiratory diseases [11 patients with cystic fibrosis (CF) and three with severe forms of bronchopulmonary dysplasia]. NMDs have the largest prevalence (79/105 subjects or 75%) with spinal muscular dystrophy (SMA) and Duchenne muscular dystrophy (DMD) being the most frequent (Figure 3). The group referred as “other NMD” included patients with neurometabolic diseases and congenital myopathies. Although contributions of each group had not changed significantly since 2010 (*p* = 0.13), relative contribution of NMD increased from 62 to 75%.

### HMV Strategy

The number of invasively and non-invasively ventilated patients was almost equal, as evident in Figures 2, 3. Even though the number of invasively ventilated subjects surpassed the number of those non-invasively ventilated since 2012, analyses did not demonstrate statistically significant difference in the number between the groups (*p* = 0.16). The invasive ventilation protocol had been initiated significantly earlier than NIV (*p* < 0.01) as seen in Table 1. NIV was performed via nasobuccal masks in 22 patients (44%) and via nasal masks (including nasal pillows) in 18 cases (36%), while the rest of the participants received a combined ventilation with both types. The combination of different masks was allowed on patient's demand in order to improve both compliance and comfort, as well as to prevent facial deformities. Unfortunately, mouthpiece ventilation has not been available for our patients.

Patients with diurnal hypoventilation and rapid progression of respiratory failure accompanied by bulbar dysfunction were continuously ventilated via tracheostomy including those with SMA type 1 and congenital myotonic dystrophy, as well as a great proportion of patients with SMA types 2/3 (7/17–41%) and DMD (4/13–30%). Patients diagnosed with NMD and neurometabolic diseases who were admitted in the ICU in an acute respiratory deterioration requiring endotracheal intubation would be eventually tracheotomized after the failure to wean.

Invasive ventilation in these subjects was in strong correlation to the lack of proper evaluation from the previous pulmonologist (*S* = 6,049, *p* < 0.01). Unfortunately, the lack of proper evaluation from the pulmonologist was the consequence of both non-attendance for arranged appointments and inadequate evaluation in regional hospitals.

NIV was performed in all others and was undertaken electively in most of the cases (44/50 or 88%). In addition, five patients initially started on NIV were eventually tracheotomized due to inadequate ventilation, while one patient with CCHS, primarily ventilated invasively, was decannulated and switched to NIV (14).

The average waiting time on equipment was counted from the day on which the decision for HMV was made up to the day when ventilation had started. It has changed significantly in recent years: 6 months of waiting until 2010 was shortened to just 1 month in 2019 (*W* = 43.5, *p* < 0.01).

The ventilator mode showed a strong correlation with the primary disease (*p* < 0.01). The pressure control mode (PC) was dominant in patients with NMDs who were invasively ventilated, while pressure support (PS) was mainly used in NIV patients with NMD and CF. Patients diagnosed with OSAS were supported by CPAP or BiPAP.

The average duration of HMV was 48 months (range 3–216 months); tracheotomized patients were ventilated longer than non-invasively ventilated ones (51 months in comparison to 45), but the difference was not statistically significant (*p* = 0.95). Comparisons among the groups revealed a significant difference in the duration of HMV between patients with OSAS and CF (*p* = 0.03), while differences between NMD and CF that were significant in 2012 were not subsequently significant (*p* = 0.21). The longest ventilated patient diagnosed with muscular dystrophy of unknown origin spent 18 years on invasive HMV, while the longest noninvasively ventilated patient diagnosed with the same primary disease has been ventilated for 15 years.

### Description of Outcomes

The number of children who died while using HMV was 25 out of the 105 (24%); 10 of them were on NIV, while 15 had been receiving invasive HMV.

A vast majority of patients died in the hospital. Four patients with CF died due to a progression of primary disease while waiting for lung transplant. Six patients with DMD died due to heart failure, four patients with numerous comorbidities died after surgical interventions, one patient died due to erosive gastritis, and one patient died due to pulmonary hemorrhage. Pneumonia was the main cause of death in six cases. Three patients died at home after the mechanical failure of the medical equipment.

Twelve patients (11%) outgrew pediatric age and have been referred to adult pulmonologists. Adherence to HMV was satisfactory, only 7 out of 105 patients (6%) included in the study had not adhered regularly and decided to stop the ventilation. With the exception of five patients who no longer needed HMV following improvement, the remaining 56 children on HMV continued to visit our center. Mortality rates in centers in other developing countries alongside general study data are summarized in Table 2.

**Table 2:** Summary of studies from developing countries.

	**Louis et al. (6)**	**Nathan et al. (9)**	**Bertrand et al. (10)**
Number of patients	55	70	35
Invasive/NIV	39/16	10/60	26/9
**Underlying disease**			
NMD	33 (60%)	7 (10%)	12 (34%)
Primary respiratory diseases	–	40 (57%)	17 (48%)
CCHS	5 (9%)	–	2 (6%)
Age at initiation of HMV (median in years)	3.5	0.9	1
Duration of HMV (median in months)	42.5	12	21
Overnight ventilation	18 (33%)^*^	38 (54%)	N/A
Ventilation 24 h a day	26 (47%)	32 (46%)	N/A
Died	21 (38%)	10 (14%)	6 (17%)

* *Information lacking for 11 patients*.

*NIV, non-invasive ventilation; NMD, neuromuscular disease; CCHS, congenital central hypoventilation syndrome; HMV, mechanical ventilation at home*.

## Discussion

After introducing modern diagnostic techniques alongside new therapeutic approaches and better overall health care, the number of ventilator-supported patients in our center increased four-fold. It is believed that this trend will continue in the future. The rapidly growing population of ventilator-supported patients, availability of the equipment, and improved care resulted in both prolongation of life and improvement of its quality. A proactive attitude led to dramatically decreased hospital expenses and hospitalization rates, particularly in NMD patients.

From the inception of the HMV program at our center, the main problem was the waiting time between clinical decision and commencement of ventilation owing to the lack of availability of the equipment. The problem was partially resolved by donations of charity organizations and the improvement of the reimbursement policy. Ventilators licensed for 24-h ventilation have increasingly become available for a selected population of patients. Reimbursement for ventilators was possible only for children with SMA and muscular dystrophies. However, the significantly increased number of ventilated patients compelled medical system authorities to improve efficacy of administrative procedures for the equipment. That was also partially enabled and facilitated by the economic growth of the country, in the last decade. According to the World Bank report, gross domestic product per capita increased from $5,674 in 2010 to $7,223 in 2018 (15)^1^ In addition, better organization in decision-making process, awareness, and support of local communities and charity donations from different sources finally resulted in substantially shortened waiting time for ventilator use.

Discharge planning for each patient started in the hospital and finalized when all prerequisites were accomplished. Primarily, the requirements were that the patient's clinical status had to be stable and necessary equipment obtained, working, and in use. Finally, caregivers had to express their capability and willingness of managing the patients at home in order for the patient to be discharged. Due to non-existence of home-care organization, home visits were not possible. A majority of the patient's needs were provided by either families or distributors of the equipment. While being hospitalized, caregivers (mostly parents) were fully trained to care for the patient at home. The training included medical procedures such as aspiration, tube feeding, cough assist techniques, basic ventilator settings and significance, and meaning of different alarms. After discharge, caregivers were informed about the possibility of contact with hospital team for any medical issue. This practice is comparable with the practices from other developing countries (9, 10).

However, a high proportion of invasively ventilated patients remain, despite all efforts to shift from invasive ventilation to NIV. In most centers across developed countries, a vast majority of patients had been ventilated noninvasively (5, 16–19), while the strategy of administrating HMV in less developed counties differs among centers (6, 9, 10). In our center, the two groups were almost even, with a slightly higher number of invasively ventilated subjects. There could be a few possible explanations for this situation.

The vast majority of tracheotomized patients were those with underlying NMDs initially admitted and intubated mostly due to respiratory infections with subsequent extubation failure. In our center, the decision about starting long-term respiratory support at home is the exclusive jurisdiction of pediatric pulmonologists. The primary goal was NIV, but in case of its failure, invasive ventilation would be initiated. A Eurovent study showed that only 24% of subjects with underlying NMD were tracheotomized (5). Most notably, all patients diagnosed with SMA type 1 and a significant proportion of subjects with SMA type 2 and DMD were invasively ventilated. While the approach of ventilation for SMA type 1 patients differs among centers, combining NIV during the night and mouthpiece ventilation during daytime had been widely recognized for respiratory support in SMA type 2 and DMD (20–24) patients. We consider timing as probably one of the main limitations; late commencement of ventilator support can be a key factor for acute respiratory deteriorations during respiratory infections leading to ICU admission and subsequent weaning failure. Nevertheless, in some patients with severe mental disability, resistant epilepsy, or bulbar insufficiency, invasive ventilation remains the best choice. Only a year after its initiation, a new oligonucleotide-based therapy (Nusinersen) for SMA patients became available in Serbia. Although useful in the very early stages of SMA, such an expensive medical therapy could be afforded only by patients in wealthier health systems, where all other prerequisites were fulfilled for efficient and safe HMV protocol administration. We are convinced that relatively inexpensive HMV equipment needs to be prioritized instead of therapies whose true benefits on respiratory outcome need to documented.

Advanced overall health care in the previous decade and initiation of the lung transplantation program since 2013 significantly improved the quality of life of the most severe patients diagnosed with CF. Initially, NIV was intended for CF patients as a bridge to transplant or as a palliative procedure, although NIV has been shown to unload respiratory muscles, improve alveolar ventilation during sleep and exercise, and facilitate the recovery of an acute exacerbation (25, 26). In recent years, we started NIV in CF patients with documented nocturnal hypoventilation, which had positive impact on preservation of respiratory function and diminished annual exacerbation rates.

There are several possible reasons for the relatively small number of OSAS patients, which is inconsistent with results of different national surveys from developed countries (18). Primarily, we speculate that the small proportion of patients with OSAS in our survey is probably caused by the inexistence of systematic national screening procedures for patients with SDB. Additionally, many patients had a combination of different contributing factors that resulted in SDB—obesity and adenotonsillar hypertrophy, in particular. Resolving one of these by adenotonsillectomy resulted in improvement of symptoms and better results of a sleep study. If one considers that CPAP/BiPAP equipment is not reimbursable, only patients with severe SDB after surgery will be started on NIV. An interesting example is the situation with patients with Prader–Willi syndrome (PWS). It is a rare disease with an estimated prevalence of 1 in 10,000 to 1 in 25,000 live births. In Serbia, annual birth rate is 50,000 newborns; thus, 2–5 new patients with PWS is expected every year. Diagnosis is mostly confirmed at infancy by genetic testing in several national tertiary pediatric hospitals. Until 2012, we had three adolescent PWS patients on BiPAP, but none in the last 7 years. In many of the PWS patients, growth hormone (GH) therapy leads to general clinical status improvement, including positive body composition changes and improvements in psychomotor development. A sleep study is recommended to assess complex patterns of SDB in each PWS patient prior to initiation of GH therapy. We can presume that a sleep study was not performed in every PWS patient, although these patients have complex patterns of SDB (27). This issue was discussed recently with other team members and hopefully will be resolved in the future.

Every 3–4 months, checkups were organized for each patient. These follow-up visits included measurements of overnight gas exchange, followed by arterial blood gases after awakening. Preset values, ventilator mode, and trigger sensitivity were carefully evaluated and eventually changed according to the results. The evaluation of patient's compliance, alarm history, and further analysis of delivered/obtained volume or pressure became possible in the last few years with technical improvements of the in-built software. A national program of palliative care in pediatrics was initiated a few years ago with the aim of sharing knowledge and experience to mobilize regional hospitals in Serbia for early diagnosis and further care and follow-up of children on HMV, in close cooperation with the national center (28).

At the end, few limitations of this study should be underlined. Firstly, a retrospective study design will lack some necessary information. In addition, it represented a relatively inhomogeneous group with a proportionately low number of patients with OSAS, because of the inexistence of systematic national screening for SDB. Finally, a small percentage of NMD patients were timely evaluated, which affects clinical practice and resulted in a high proportion of patients having been ventilated invasively.

## Conclusion

In the last decade, our national HMV program has been conducted successfully, despite numerous obstacles during that period. Both availability and shortened waiting time for modern ventilator equipment accompanied by advanced overall health care are basics of substantial improvement. With our experience, in a relatively small developing country such as Serbia, one well-organized HMV center is adequate. A well-developed national network of regional hospitals that spreads knowledge and exchanges experiences with other pediatric pulmonologists is of great importance. In future, improvements could be directed to emphasize the importance of early diagnosis of SDB and nocturnal hypoventilation as an early sign of respiratory failure to avoid acute deterioration and potential subsequent invasive ventilation. We also hope that equipment for HMV will be available to all the affected children, regardless of primary cause of disease.

## Data Availability Statement

The datasets generated for this study are available on request to the corresponding author.

## Ethics Statement

The studies involving human participants were reviewed and approved by Ethical Committee of The Institute for Health Protection of Mother and Child of Serbia. Written informed consent to participate in this study was provided by the participants' legal guardian/next of kin.

## Author Contributions

MB contributed in literature search, data collection and organization of database, study design, statistical analysis, manuscript preparation, and manuscript revision. PM contributed in statistical analysis, manuscript preparation, and manuscript revision. MR contributed in data collection and organization of database, study design, and manuscript revision. AS contributed in literature search, data collection and organization of database, study design, statistical analysis, manuscript preparation, and manuscript revision.

## Conflict of Interest

The authors declare that the research was conducted in the absence of any commercial or financial relationships that could be construed as a potential conflict of interest.

## Footnotes

^1^https://www.worldbank.org.

## References

[1] Amin RS Fitton CM. Tracheostomy and home ventilation in children. Semin Neonatol. (2003) 8:127–35. 10.1016/S1084-2756(02)00220-8 15001149

[2] Ottonello G Ferrari I Pirroddi IM Diana MC Villa G Nahum L. Home mechanical ventilation in children: retrospective survey of a pediatric population. Pediatr Int. (2007) 49:801–5. 10.1111/j.1442-200X.2007.02463.x 18045275

[3] Hammer J. Home mechanical ventilation in children: indications and practical aspects. Schweiz Med Wochenschr. (2000) 130:1894–902. 11153395

[4] Wallis C Paton JY Beaton S Jardine E. Children on long-term ventilatory support: 10 years of progress. Arch Dis Child. (2011) 96:998–1002. 10.1136/adc.2010.192864 21109507

[5] Lloyd-Owen SJ Donaldson GC Ambrosino N Escarabill J Farre R Fauroux B. Patterns of home mechanical ventilation use in Europe: results from the Eurovent survey. Eur Respir J. (2005) 25:1025–31. 10.1183/09031936.05.00066704 15929957

[6] van der Poel LAJ Booth J Argent A Van Dijk M Zampoli M Home ventilation in South African children: do socioeconomic factors matter? Pediatr Allergy Immunol Pulmonol. (2017) 30:163–70. 10.1089/ped.2016.0727 35923010

[7] Oktem S Ersu R Uyan ZS Cakir E Karakoc F Karadag B. Home ventilation for children with chronic respiratory failure in Istanbul. Respiration. (2008) 76:76–81. 10.1159/000110801 17984626

[8] Nasiłowski J Wachulski M Trznadel W Andrzejewski W Migdał M Drozd W. The evolution of home mechanical ventilation in Poland between 2000 and 2010. Respir Care. (2015) 60:577–85. 10.4187/respcare.03126 25492950

[9] Nathan AM Loo HY de Bruyne JA Eg KP Kee SY Thavagnanam S. Thirteen years of invasive and noninvasive home ventilation for children in a developing country: a retrospective study. Pediatr Pulmonol. (2017) 52:500–7. 10.1002/ppul.23569 27712049

[10] Bertrand P Fehlmann E Lizama M Holmgren N Silva M Sánchez I. Home ventilatory assistance in Chilean children: 12 years' experience. Arch Bronconeumol. (2006) 42:165–70. 10.1016/S1579-2129(06)60437-0 16735012

[11] Sovtic A Minic P Vukcevic M Markovic-Sovtic G Rodic M Gajic M. Home mechanical ventilation in children is feasible in developing countries. Pediatr Int. (2012) 54:676–81. 10.1111/j.1442-200X.2012.03634.x 22462757

[12] Paiva R Krivec U Aubertin G Cohen E Clément A Fauroux B. Carbon dioxide monitoring during long-term noninvasive respiratory support in children. Intensive Care Med. (2009) 35:1068–74. 10.1007/s00134-009-1408-5 19172246

[13] Amaddeo A Moreau J Frapin A Khirani S Felix O Fernandez-Bolanos M. Long term continuous positive airway pressure (CPAP) and noninvasive ventilation (NIV) in children: initiation criteria in real life. Pediatr Pulmonol. (2016) 51:968–74. 10.1002/ppul.23416 27111113

[14] Paglietti MG Porcaro F Sovtic A Cherchi C Verrillo E Pavone M. Decannulation in children affected by congenital central hypoventilation syndrome: a proposal of an algorithm from two European centers. Pediatr Pulmonol. (2019) 54:1663–69. 10.1002/ppul.24448 31313536

[15] World Bank Group (2018). Internet page of World Bank. Available online at: https://www.worldbank.org

[16] Racca F Berta G Sequi M Bignamini E Capello E Cutrera R. Long-term home ventilation of children in Italy: a national survey. Pediatr Pulmonol. (2011) 46:566–72. 10.1002/ppul.21401 21560263

[17] Ikeda A Tsuji M Goto T Iai M. Long-term home non-invasive positive pressure ventilation in children: results from a single center in Japan. Brain Dev. (2018) 40:558–65. 10.1016/j.braindev.2018.03.006 29636207

[18] Fauroux B Boffa C Desguerre I Estournet B Trang H. Long-term noninvasive mechanical ventilation for children at home: a national survey. Pediatr Pulmonol. (2003) 35:119–25. 10.1002/ppul.10237 12526073

[19] Pavone M Verrillo E Onofri A Caggiano S Chiarini Testa MB Cutrera R. Characteristics and outcomes in children on long-term mechanical ventilation: the experience of a pediatric tertiary center in Rome. Ital J Pediatr. (2020) 46:12. 10.1186/s13052-020-0778-8 32005269 PMC6995086

[20] Simonds AK Ward S Heather S Bush A Muntoni F. Outcome of paediatric domiciliary mask ventilation in neuromuscular and skeletal disease. Eur Respir J. (2000) 16:476–81. 10.1034/j.1399-3003.2000.016003476.x 11028663

[21] Katz S Selvadurai H Keilty K Mitchell M MacLusky I. Outcome of non-invasive positive pressure ventilation in paediatric neuromuscular disease. Arch Dis Child. (2004) 89:121–4. 10.1136/adc.2002.018655 14736624 PMC1719799

[22] Wang CH Finkel RS Bertini ES Schroth M Simonds A Wong B. Consensus statement for standard of care in spinal muscular atrophy. J Child Neurol. (2007) 22:1027–49. 10.1177/0883073807305788 17761659

[23] Bach JR. The use of mechanical ventilation is appropriate in children with genetically proven spinal muscular atrophy type 1: the motion for. Paediatr Respir Rev. (2008) 9:45–50. 10.1016/j.prrv.2007.11.003 18280979

[24] Ryan MM. The use of invasive ventilation is appropriate in children with genetically proven spinal muscular atrophy type 1: the motion against. Paediatr Respir Rev. (2008) 9:51–4. 10.1016/j.prrv.2007.10.002 18280980

[25] Fauroux B Nicot F Essouri S Hart N Clément A Polkey MI. Setting of noninvasive pressure support in young patients with cystic fibrosis. Eur Respir J. (2004) 24:624–30. 10.1183/09031936.04.0000137603 15459142

[26] Fauroux B. Why, when and how to propose noninvasive ventilation in cystic fibrosis? Minerva Anestesiol. (2011) 77:1108–14. 21602746

[27] Pavone M Caldarelli V Khirani S Colella M Ramirez A Aubertin G. Sleep disordered breathing in patients with Prader-Willi syndrome: a multicenter study. Pediatr Pulmonol. (2015) 50:1354–9. 10.1002/ppul.23177 25851435

[28] Rusalen F Agosto C Brugnaro L Benini F. Impact of the regional pediatric palliative care network on the care of children on long-term ventilation: could the availability of a residential solution into the network reduce the duration of intensive care unit staying for these patients? J Pediatr Intensive Care. (2018) 7:75–80. 10.1055/s-0037-1605369 31073474 PMC6260342

